# Transcatheter mitral valve repair for primary mitral regurgitation

**DOI:** 10.31083/j.rcm2304116

**Published:** 2022-03-26

**Authors:** Rowa H. Attar, Stephen H. Little, Nadeen N. Faza

**Affiliations:** ^1^Department of Cardiology, Houston Methodist DeBakey Heart & Vascular Center, Houston, TX 77030, USA

**Keywords:** mitral regurgitation, mitraClip, transcatheter mitral valve repair

## Abstract

The landscape of transcatheter mitral valve repair devices continues to expand, with many technologies undergoing investigation in patients with primary mitral regurgitation (MR). Transcatheter edge-to-edge repair (TEER) of the mitral valve is currently approved for management of patients with severe primary MR who are deemed to be high risk surgical candidates. The current review will focus on an integrative clinical and echocardiographic approach to guide patient selection, intra-procedural imaging guidance, and post procedural follow up in patients undergoing TEER. This review will also highlight future directions in transcatheter repair techniques of the mitral valve.

## 1. Introduction

Primary Mitral regurgitation (MR) is instigated by a primary anomaly of the mitral apparatus. This may be due to idiosyncrasy of the leaflets, chordae tendineae or papillary muscles. Severe MR inadvertently leads to a chronic volume overload state resulting in left ventricular dilatation and dysfunction and has been associated with a poor outcomes and portentous prognosis. Intervention in a judicious manner may result in alteration of clinical trajectory and advantageous clinical course. Thus, the importance of early diagnosis, precise delineation of etiology and timely intervention in patients presenting with severe primary MR cannot be overemphasized [1]. Transcatheter edge-to-edge repair has been proven as a safe and effective technique to treat Primary MR with culminating clinical evidence proving durability and efficiency.

The purpose of the current review is to outline an integrative clinical and echocardiographic approach to diagnose primary MR with a focus on appropriate patient selection, intraprocedural guidance and post procedure follow up for patients undergoing transcatheter edge-to-edge repair (TEER) of the mitral valve. This review will also highlight future directions in transcatheter repair techniques of the mitral valve.

**Fig. 1.**
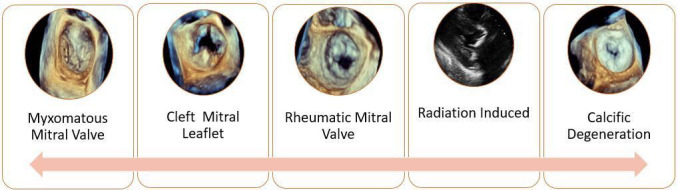
**Variable etiologies of primary mitral regurgitation**. The figure demonstrates the spectrum of mitral valve pathology. The first image is a 3D image of a Barlow’s mitral valve showing billowing of the mitral leaflets. The Second image is 3D image of the mitral valve with a cleft of the anterior mitral leaflet seen at the 12 O’Clock position. The third is image is a 3D echocardiographic representation of a rheumatic mitral valve showing fusion of both medial and lateral commissures. The fourth image is a 2D image of radiation induced mitral valve disease showing severe thickening and calcification of the mitral annulus and mitral valve leaflets with involvement of the aorto-mitral curtain. Lastly the image farthest to the right is a 3D image of the mitral valve showing severe calcification and degeneration.

Primary MR is defined as a predominant pathology of the mitral valve apparatus. The leading etiology of primary MR is myxomatous degeneration of the mitral valve leaflets leading to mitral valve prolapse [2]. Myxomatous degeneration can result from a continuum of clinical presentations ranging from a more subtle presentation of patients with fibroelastic deficiency resulting in chordal rupture and flail leaflets in older individuals to more extensive phenotypes with Barlow’s disease and diffusely thickened and redundant leaflets [3]. Other etiologies of primary MR include primary leaflet perforation, cleft leaflets, rheumatic disease or drugs such as ergotamine, cabergoline and 3,4-methylenedioxymethamphetamine (MDMA, also known as ecstasy). Restricted leaflet motion and thickening of leaflet edges and sub valvular apparatus can also result from therapeutic radiation and long-standing connective tissue disease. Both conditions can lead to clinically significant MR that is difficult to treat. With the prevalence of an aging population globally, there has been note of a degenerative process that begins in the posterior annulus and extends to the base of the leaflets and sub valvular apparatus. This process has been increasingly recognized as a challenging process that affects annular and leaflet function [4]. Determining the exact etiology of primary MR is key in patient selection for transcatheter mitral valve procedures [5]. Fig. 1 illustrates echocardiographic morphologies of different mitral valve pathologies.

Neglected severe primary MR has been associated with poor outcomes in early studies. The presence of a flail leaflet resulting in severe MR was observed to be an adverse prognostic feature. Patients with this finding in one study, had either required surgery or were deceased at 10 year follow up [6]. Other markers of poor prognosis were the incidence of atrial fibrillation and heart failure. Both findings were independently associated with reduced survival. In patients with asymptomatic severe MR, one prospective observational study reported an increase in both mortality and cardiac events with increasing degree of regurgitation. One echocardiographic marker, the effective regurgitant orifice area of more than 40 mm in this study correlated with poorest outcome [7].

TEER is a percutaneous replication of the surgical edge-to-edge repair developed by Alfieri [8, 9, 10] in the early 1990s to treat MR. The surgical procedure consists of creating a valve with two orifices by suturing the free edge of the leaflets at the origin of the regurgitation. Historically this was commonly done on the middle A2-P2 scallops. The edge-to-edge technique was first performed in 1991 to successfully treat a patient with anterior leaflet prolapse. Most patients would undergo mitral valve replacement due to challenges associated with repair of the anterior mitral leaflet. The hemodynamic effects of this procedure were questioned due to concerns of the hemodynamic effects a double orifice may cause. Multiple reports observed the hemodynamic and anatomic effects of this technique [11, 12]. The concern was the risk of creating mitral stenosis, although this was rarely seen in clinical practice. A virtual model of the double orifice mitral valve with orifices of comparable or dissimilar dimension advocated hemodynamics were not affected by the double orifice conformation, even when the double orifice suture was disproportionate or when this led to distortion of the valve [13]. More recently, observational studies [14] demonstrated that the double orifice technique does not alter valve diastolic function either at rest or under exercise. Clinical studies corroborate good long-term outcomes in patients treated surgically without annuloplasty. Some reports have shown that in select patients’ durability was as long as 12 years [15, 16].

TEER of the mitral valve based on this surgical method was developed by the use of a clip with grasping arms rather than a suture to secure the mitral leaflets [17, 18]. The trans-septal approach was used to deliver a clip device that can grasp the mitral leaflet edges to create a double orifice.

In 2006, Feldman *et al*. [17] reported 6-month outcomes of a phase I feasibility and Safety study of the MitraClip (Abbott, Chicago, IL, USA) device in patients with hemodynamically significant primary MR (EVEREST). This study demonstrated safety and effectiveness of the MitraClip device in MR reduction in appropriately selected patients. In 2011, the EVEREST II trial was published [18]. EVEREST II was a randomized trial that compared TEER to mitral valve surgery in a 2:1 randomization. 279 patients with moderately severe and severe primary MR were enrolled [18]. The primary effectiveness outcome favored surgery at one year due to greater reduction in MR. Despite the latter observation, patients who underwent TEER had significantly reduced left ventricular end-diastolic volume and dimensions, improved New York Heart Association (NYHA) functional class, and improved quality of life at 12 months, as compared with baseline measures [18]. In 2015, Feldman *et al*. [19] reported five-year outcomes of the EVEREST II trial. Five year follow up results of this randomized trial confirmed initial 12 months findings of superior MR reduction with surgery, however patients that had undergone TEER were found to have durable reduction in MR along with reverse Left ventricular(LV) remodeling and a sustained improvement in symptoms and quality of life despite substandard initial MR reduction post procedurally. Quantitatively less MR reduction translated to clinically meaningful hemodynamic and clinical outcomes. Follow up results also provided further safety data and need for redo TEER or surgical intervention was scarce.

The most recent iteration of the American Heart Asscociation/American College of Cardiology AHA/ACC valve guidelines published in 2020 by Otto *et al*. [20] propose that TEER is reasonable in patients with severe symptomatic (NYHA III-IV) primary MR and high or prohibitive surgical risk. The 2021European Society of Cardiology/European Association of Cardiothoracic Surgery ESC/EACTS guidelines for the management of valvular heart disease also give a similar recommendation whereby TEER may be considered in patients who fulfill echocardiographic criteria of eligibility and found to be at high or prohibitive surgical risk by the heart team [21]. Both consensus statements emphasize the importance of assessing overall life expectancy with specific recommendation of TEER in patients who have an anticipated life expectancy of more than one year [22, 23, 24, 25].

## 2. Baseline evaluation

The initial test recommended by consensus guidelines for evaluation of MR to establish mechanism, severity and resultant hemodynamic sequelae is a transthoracic echocardiogram (TTE) [20]. The most recent recapitulation of the American Society of Echocardiography (ASE) native valve regurgitation guidelines by Zoghbi *et al*. [26] and the ASE Transesophageal echocardiography (TEE) guidelines for screening for structural heart guidelines published by Hahn *et al*. [27] recommend the following baseline measures prior to intervention to help guide appropriateness and procedural planning if indicated. 2D parameters such as chamber size and function, along with delineation of valve anatomy and identification of flail segments, prolapse or perforation are usual starting points. Other helpful parameters to help quantitate lesion severity include color flow Doppler assessment using jet flow density, proximal flow convergence, vena contracta and proximal isovelocity surface area (PISA). In addition, pulse wave doppler parameters such as mitral inflow pattern and pulmonary vein flow pattern also provide crucial information to help quantitate regurgitation severity [26].

The role of TEE in assessing the mechanism of MR, quantitating severity of regurgitation and in determining candidacy for TEER is central to any pre-procedural assessment. The use of 3D TEE provide detailed anatomical and functional assessment of the mitral valve leaflets. TEE images provide imperative information to both the structural imager and interventional cardiologist [27]. An example of such data points inlcude a 3D mitral valve area, leaflet length and mitral annulus to fossa height. Identifying precise leaflet pathology, location and mechanism is fundamental in appropriate patient selection, device choice and necessary in creating a prodeural roadmap for the interventional cardiologist in determing clip deployment strategy [28, 29].

### 2.1 Patient selection

The EVEREST II clinical trial used the original MitraClip device, which is no longer commercially available [17, 18]. Augmentation of MitraClip features and nuanced clinical experience with MitraClip has resulted in expansion of mitral selection criteria beyond the initial inclusion criteria used in (EVEREST II) [30, 31]. Table 1 demonstrates anatomical features that favor feasibility of TEER.

**Table 1.** **Role of baseline imaging in determining feasibility of TEER**.

Anatomic features favoring feasible repair	
	A2/P2 Prolapse
	Flail A2/P2 with a flail gap less than 10 mm and flail width less than 15 mm
	Single central jet
	Trans-septal crossing height to mitral annulus plane >4 mm
	Non-tethered leaflets and leaflet length of more than 10 mm
	Baseline mitral valve gradient less than 3 mmHg
Predictors of challenging or suboptimal procedural outcomes	
	Commissural Prolapse
	Barlow’s mitral valve
	Anterior leaflet prolapse with ruptures chordae
	Multiple prolapse segments
	Multiple flail segments
	Cleft at or adjacent to leaflet grasping area
	Post mitral annuloplasty repair
	Severe mitral annular calcification (<5 mm leaflet available for grasping)
	Leaflet or chordal calcification
	Mobile posterior leaflet length less than 7 mm
	Tethering height more than 11 mm
	Planimetered mitral valve area less than 4 ${\mathrm{cm}}^{2}$ and mitral valve mean gradient 4–5 mmHg
	Small left atrial size (medial – lateral diameter <3.7 cm)
	Lipomatous interatrial septum, patent foramen ovale or previous surgical or device closure
Features suggesting prohibitive risk	
	Left atrial/atria appendage thrombus
	Calcification of leaflets in grasping zone
	Mitral valve Mean gradient >5 mmHg
	Severe right ventricular dysfunction and pulmonary hypertension unrelated to valve disease
	Interatrial septal occluder device that cannot be crossed with transcatheter electrocautary

### 2.2 Food and Drug Admins (FDA) approved devices

The MitraClip device (Abbott) is a commercially available device used for TEER in patients with significant symptomatic primary MR. The device was initially approved by the United States Food and Drug Administration (FDA) on October 2013 based on results of the EVEREST II study of patients with primary MR who are at a high risk for surgery. The first in human MitraClip implant was performed in 2003 with the original MitraClip NT device [32, 33, 34]. Since then, the device and delivery system have undergone several technical advances to enhance clip delivery and treat more diverse mitral pathologies [35].

The most novel MitraClip generation is the MitraClip G4 system. Fundamental characteristics of the MitraClip G4 include the capacity to detect left atrial pressure during the procedure, both independent and simultaneous gripper actuation, and the availability of two new clip sizes: some leading features of the newer generation MitraClip include having a wider grasping surface allowing for more grasp of the flail segment within clip arms [36]. Chakravarty *et al*. [37] reported outcomes of 59 patients in which the MitraClip G4 system was used. High safety and efficacy of the G4 system, with 96.5% of patients having reduction in MR grade to 2+ at 30 days were reported. Garcia-Sayan *et al*. [38] also reported similar outcomes in their cohort of 61 patients. They reported procedural success rate of 96.7% and technical failure rate of 1.6%. Their cohort was inclusive of complex mitral pathology including prior mitral valve repair, multi-scallop and commissural pathology. The observational EXPAND G4 is a post market study that will assess safety and performance of of the MitraClip G4 System and this registry will evaluate clinical and echocardiographic outcomes with the MitraClip G4 system [39]. Table 2 illustrates the different FDA approved TEER devices with salient features of MR that would aid in clip selection.

**Table 2.** **Device Selection for TEER based on anatomical features of the mitral valve and mitral regurgitant jet**.

Clip type	NT	NTW	XT	XTW
Arm length	9 mm	9 mm	12 mm	12 mm
Arm Width	4 mm	6 mm	4 mm	6 mm
Select Considerations	Borderline MVA (3.5–4 ${\mathrm{cm}}^{2}$)	Secondary MR with a wide elongated jet	Adjunct to XTW for additional MR reduction if there is concern about MVA	Preferred for primary MR with large flail or bileaflet prolapse (flail width >15 mm)
	Narrow Circular jet			
	Fail width <15 mm			
	Commissural Pathology		Central A2-P2 pathology	
	Short or restricted PML (6–9 mm)		Long or redundant PML >9 mm	
	Mitral Annular Calcification		Absence of mitral annular calcification	
	Coaptation/Flail Gap <10 mm		Large coaptation gap or height	

**Fig. 2.**
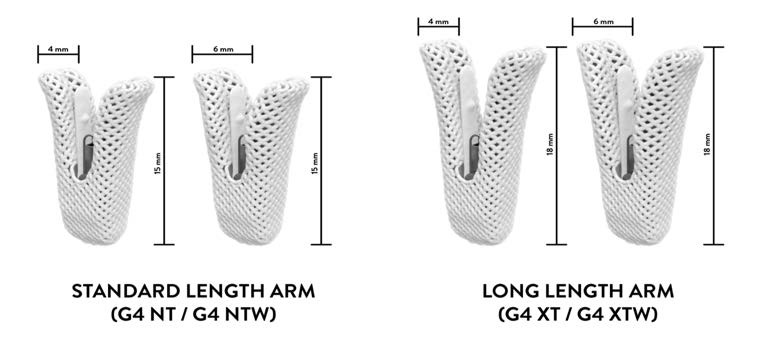
**Comparison of MitraClip G4 Dimension**. The figure on the left shows standard length MitraClip NT and NTW dimensions and the right image shows the MitraClip G4 XT and XTW dimensions demonstrating longer arm length.

**Fig. 3.**
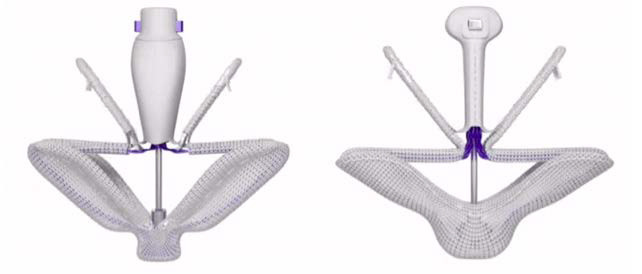
**Configuration of the PASCAL and PASCAL Ace devices**. The left image shows the PASCAL device with two independent clasps and two paddles that allow leaflet grasping. A central spacer is seen in both devices. The central spacer is intended to fill the regurgitant orifice. The PASCAL Ace device is shown on the right image with similar configuration.

Fig. 2 illustrate feature and dimensions of the MitraClip System and Fig. 3 illustrate features and dimensions of the PASCAL system.

## 3. Intraprocedural guidance

The first step in TEER is acquisition of baseline images to determine the etiology of MR with precise localization of the scallops involved. Baseline images are also obtained to exclude contraindications to TEER. Important pathologies to exclude are the presence of left atrial thrombi and the presence of vegetations suggestive of active infective endocarditis [28].

Intraprocedural guidance involves identification of landmark structures such as the interatrial septum (IAS), left atrium, left atrial appendage and left superior pulmonary vein.

An enface 3-dimensional (3D) view of the mitral valve, also known as the surgeon’s view, is utilized as the default view to facilitate communication between the interventionalist and the structural heart disease imager. TEER of the mitral valve involves the following procedural steps:

-Transseptal puncture:

The ideal transseptal puncture site for TEER is in the superior and posterior aspect of the fossa ovalis, 4–5 cm above the mitral valve annulus. Medial pathologies require a higher transseptal height as compared to lateral pathologies. Transseptal height in patients with primary MR would ideally also require additional height in comparison to patients with functional MR. Patients with functional MR typically present with a dilated mitral annulus, apical tenting of the mitral leaflets and tethering, downward displacement of the mitral leaflets allows for such pathologies to be treated even if the height from annulus to fossa is less than 4 cm. In patients with functional MR height needed for transseptal puncture can be taken from site of pathology to the fossa. A short-axis view at the level of the aortic valve helps identify the anterior aspect of the septum that is closest to the aortic valve. A bicaval view identifies the superior and inferior aspect of the interatrial septum adjacent to the superior and inferior vena cava, respectively. Biplane imaging, which provides 2 orthogonal views of the septum, can be used to simultaneously confirm the superior and posterior location of the puncture.

-Advancement of the Steerable Guide Catheter:

After transseptal puncture is performed, a wire is passed into the left upper pulmonary vein followed by a dilator across septum. The steerable guide catheter (SGC) is then advanced into the left atrium. 2D and 3D TEE imaging of the septum and left atrium allow for visualization of the SGC and its trajectory in the left atrium.

-Positioning of the Device:

The MitraClip device is then advanced through the SGC into the left atrium. Imaging is key in determining the device location in relation to the coumadin ridge, roof of the atrium, and the left upper pulmonary vein. Once the device is positioned proximal to the coumadin ridge, it is redirected into the mitral valve annulus perpendicular to site of pathology. 3D TEE imaging plays a key role in positioning the device during this key step. The device can be moved in a medial, lateral, anterior, or posterior direction and can additionally be rotated to ensure perpendicular alignment with the coaptation line and target pathology. Precise positioning of the device is important to minimize maneuvering of the device in the left ventricle to prevent chordal entanglement.

-Leaflet grasping:

After the device is accurately positioned in the left atrium, the device is closed and advanced into the left ventricle. The grasping view by TEE where the long arms of the device are visualized is the long axis view at approximately 120 degrees. This can vary based on the patient’s anatomy and the site of pathology. Clear visualization of the leaflets as they rest deep in device arms is important to make sure enough tissue is being grasped. The grippers are then dropped, and the device is then closed.

-Hemodynamic Assessment:

Assessment of residual MR relies on a multiparametric approach. Echocardiographic signs of MR reduction include a reduction in the size of the regurgitant jet, which can be challenging in the presence of multiple or eccentric jets. Other parameters include an increase in left ventricular outflow tract stroke volume (by trans gastric imaging), presence of spontaneous echocardiographic contrast in the left atrium, and improvement in the pulmonary venous systolic flow. A study by Avenatti *et al*. [40] showed that a 3D vena contacta area threshold of 0.27 ${\mathrm{cm}}^{2}$ has a good diagnostic accuracy for identification of ≥ moderate MR.

If MR reduction is not satisfactory, repositioning the device or adding a second device is needed, in the absence of mitral stenosis. Before a device is deployed, assessment of tans-mitral gradients are performed to ensure that the device has not resulted in significant mitral stenosis.

**Fig. 4.**
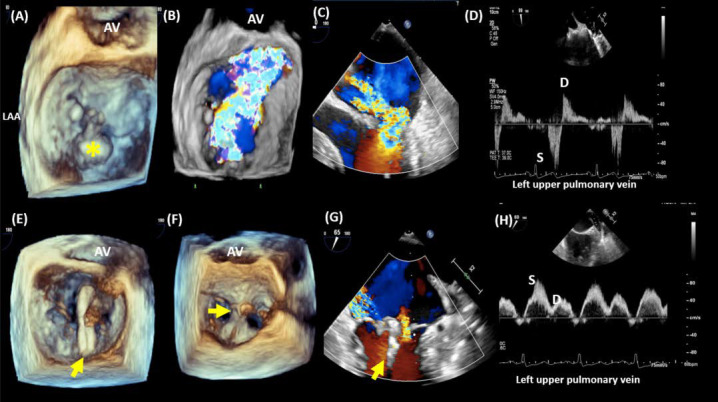
**Transcatheter edge-to-edge repair of a case of a flail posterior leaflet**. 3-dimensional imaging (A) demonstrates a flail P2 (*) with significant anteriorly directed MR (B). 2-dimensional baseline TEE imaging demonstrates the flail posterior leaflet with severe anteriorly directed MR (C). Left upper pulmonary venous flow at baseline shows systolic flow reversal, indicative of severe MR (D). The MitraClip device (arrow) is advanced into the left atrium and positioned perpendicular to the line of coaptation of A2-P2 (E). The device is then advanced into the LV and deployed to approximate the A2-P2 leaflets, resulting in a tissue bridge (F). This results in significant reduction in MR (G) with dominant systolic flow in the left upper pulmonary venous flow (H). AV, aortic valve; LAA, left atrial appendage.

Fig. 4 demonstrates successful TEER of the mitral valve in a patient with P2 leaflet flail.

## 4. Detection of complications

The Mitral Valve Academic Research Consortium (MVARC) standardized the endpoint and complications definitions for transcatheter mitral valve repair in 2015 [41, 42]. Complications may be broadly categorized to procedure-related events and device associated events. Procedure-related complications mainly result from vascular access and transseptal puncture. Transseptal puncture is a safe procedure with a reported major complication rate ranging between 1–2% when performed under echocardiography guidance [43, 44].

A rare complication of TEER include perforation of cardiac chamber or great vessel [45]. This dreaded complication has been reported to be less than 2% in some studies [46]. This will occasionally result from a misdirected transseptal puncture. Patients that have large atria, thick or redundant septum and prior surgery involving the atrial septum are at higher risk of septal complications. In the inadvertent event of aortic perforation, it must be cautioned that if a delivery sheath or catheter has been advanced, the catheter should not be withdrawn prematurely. The most reasonable strategy to contain this complication would include immediate surgical intervention while pericardiocentesis with concomitant autotransfusion maybe used as a temporizing measure awaiting surgery [43].

Multiple trials and registries have repeatedly demonstrated safety of the MitraClip [30, 42]. Initial pivotal trials reported a complication rate ranging between 0 to 4.3% [30].

**Fig. 5.**
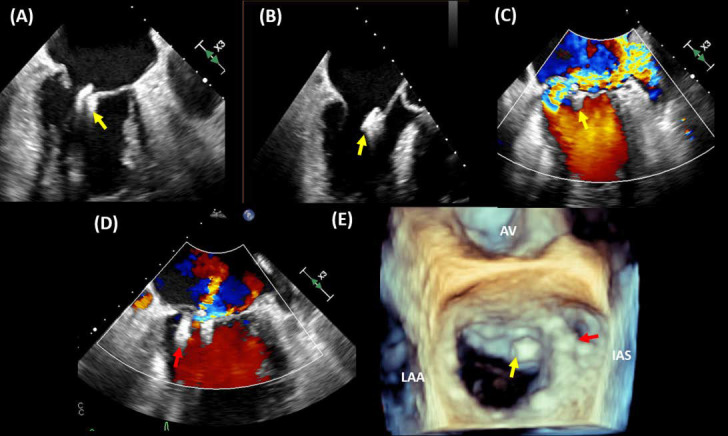
**Single leaflet detachment**. A case of single leaflet device attachment (SLDA). A patient with previous transcatheter edge-to-edge repair of the mitral valve presents with heart failure symptoms and TEE imaging showing SLDA of the MitraClip device (yellow arrow). The device was attached to the anterior leaflet (A&B) with severe mitral regurgitation (C). A second MitraClip device (red arrow) was deployed medial to the first device, resulting in mild residual MR (D). 3 TEE imaging shows the newly implanted device at A3-P3 in relation to the first device that has detached from the posterior leaflet (E). AV, aortic valve; LAA, left atrial appendage; IAS, interatrial septum.

Single leaflet device attachment (SLDA) describes and entity in which there is disengagement of insertion of one of the leaflets from the MitraClip device and can occur in 2–5% of cases. This entity can occur during the procedure or follow-up [17, 18, 42, 45]. One of the most important tasks of the structural heart imager entails the acquisition of high-resolution grasping views that display leaflet insertion into clip arms ensuring a good grasp with both leaflets tucked in the closed device. Some strategies to stabilize SLDA include the deployment of additional clips if feasible [40, 47, 48]. Fig. 5 shows TEE imaging demonstrating the attachment of the clip to a single leaflet.

Clip embolization is defined as device detachment from both leaflets during or after the procedure and occurs in less than 1% [47, 48, 49, 50]. The clip may travel to distal arteries causing ischemia. Surgical removal in this dreaded complication is usually required.

**Fig. 6.**
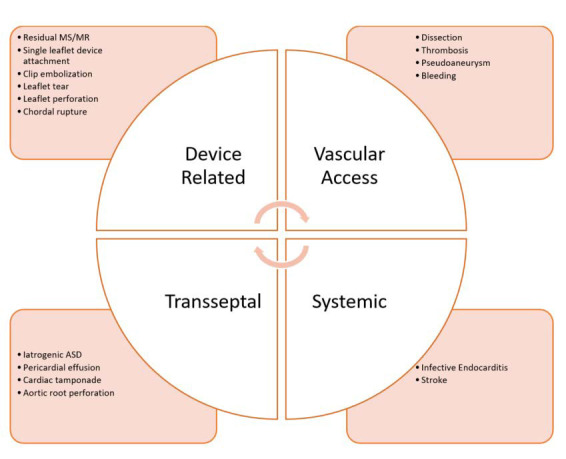
**Potential complications of transcatheter edge to edge repair of the mitral valve**. Visual representation of different potential complications that may be associated with TEER.

The current literature suggests there may be a rate of spontaneous closure of iatrogenic atrial septal defect (ASD) over long-term clinical follow up [51, 52, 53, 54, 55]. One study eluded to a correlation between the persistence of an iatrogenic atrial septal defect and elevated left atrial pressures after clip deployment [56]. The clinical impact of an iatrogenic atrial septal defect remains an area of debate with multiple studies suggesting persistence of an iatrogenic atrial septal defect to be associated with an increase in mortality and rehospitalization rate after TEER [51, 54, 57, 58]. While other data reported by Hoffman *et al*. [59] was suggestive of a positive hemodynamic effect with iatrogenic ASD in patients post TEER. Fig. 6 outlines potential complications associated with TEER.

## 5. Follow up

The ASE Consensus guidelines for assessment of MR post TEER recommend that a transthoracic echocardiogram be performed on the first post procedural day, at 30 days and at 6–12 months [60, 61, 62]. The immediate post procedure follow-up study is done to assess procedural outcomes and rule out acute complications [63]. The purpose of the echo that is done at the 6–12 months mark aims to define longterm hemodynamic effects of MR reduction such as favorable reverse remodeling of the left ventricle and left atrium [64, 65, 66, 67, 68, 69], and possible decrease in pulmonary artery pressure. Assessment of residual MR remains a challenging area that entails further study. MR grading may be difficult due to the complexity of its mechanisms after TEER, the frequent multiple eccentric jets of variable sizes, and shadowing from the devices. Color flow Doppler continues to be the initial screening tool for severity assessment. Evaluation of residual MR requires careful integration of multiple parameters, as no single parameter is sufficiently accurate to assess MR severity. The use of the PISA method for MR quantitation is not advised after TEER [60]. In certain scenarios, cardiac magnetic resonance imaging may be of potential benefit when more than mild MR is suspected as it likely has the advantage of calculating regurgitant volume and fraction and may provide a comprehensive estimate of severity [68]. Table 3 highlights echocardiographic parameters used to assess residual MR post TEER [60].

**Table 3.** **Echocardiographic parameters used to assess residual mitral regurgitation after TEER**.

Echocardiographic parameter	Mild MR	Moderate MR	Severe MR
Device position	Appropriate position/normal motion	No specific criteria	Abnormal device position, flail or detachment seen
LA/LV Volumes	Reduction in size from baseline	Minimal change	Enlarged/worsening from baseline
Color Doppler	One or two small narrow jets	More than mild but does not meet severe criteria	Large central/multiple jets/eccentric jet of any size with wrap around LA
Flow Convergence	None or small	Intermediate	Large
Mitral Inflow	A-Wave dominant	No specific pattern	No specific pattern
Pulmonary Vein flow	S wave dominant	Blunted systolic flow	Systolic flow reversal
CW Doppler of MR	Faint parabolic	No specific criteria	Dense triangular contour
Vena Contracta	Single jet VCW <0.3 cm	Single jet VCW 0.4 cm–0.6 cm	Jet width >0.7 cm or more than two moderate jets
Vena Contracta area by 3D	VCA <0.2 ${\mathrm{cm}}^{2}$	VCA 0.2–0.39 ${\mathrm{cm}}^{2}$	VCA >0.4 ${\mathrm{cm}}^{2}$ or >2 moderate jets
Regurgitant Volume	<30 mL	30–60 mL	>60 mL (may be lower in low flow states)
Regurgitant Fraction	<30%	30–49%	>50%

Note: *Adapted from “Recommendations for Noninvasive Evaluation of Native Valvular Regurgitation. A Report from the American Society of Echocardiography Developed in Collaboration with the Society for Cardiovascular Magnetic Resonance” By W.A. Zoghbi.* Copyright 2017 by the American Society of Echocardiography. http://dx.doi.org/10.1016/j.echo.2017.01.007

## 6. Challenging septal anatomies

Variations in septal anatomy are prevalent. The etiologies of variable septal orientation may be secondary to extracardiac or intracardiac reasons. Some of the most commonly encountered reasons to cause distortion to septal alignment are factors that alter the cardiac axis within the chest wall such as an increase in abdominal girth, chronic obstructive lung disease and tortuosity of the aorta related to aging. In the presence of extreme dilation of the aorta the interventional imager and proceduralist should exercise extreme care in avoidance of an anterior septal puncture as this may result in aortic injury. Other causes of extracardiac septal variation includes spinal disorders such as scoliosis and kyphosis. Dilatation of the cardiac chambers such as extreme left atrial dilation may also lead to distortion of the interatrial septal position.

Some innate abnormalities of the interatrial septum include the presence of lipomatous hypertrophy, a floppy and redundant septum, atrial septal aneurysm, fibrosis of the septum and the presence of an atrial septal closure device. In the presence of a septal aneurysm use of a Safe Sept wire may be advised and avoidance of excessive tenting to prevent inadvertent crossing and injury to the free left atrial wall. When encountering a fibrosed interatrial septum wire mediated crossing should be considered or radiofrequency ablation when necessary. Lastly in the presence of an atrial septal occluder device, multimodality imaging may be required to determine if there is remaining fossa ovalis rim that can be safely traversed surrounding the device. A retrospectively gated cardiac computed tomography would be especially helpful in such scenario. Most closure devices can be crossed in the absence of residual rim with careful planning and consideration of post procedural closure if needed.

## 7. Challenging mitral anatomy

Barlow’s disease with multisegmented prolapse poses complex technical challenge to TEER. The majority patients with Barlow’s tolerate MR for decades and will only become symptomatic in the presence of a concomitant flail segment or chordal rupture. Delayed presentation may occur in the 8th and 9th decade making surgical risk prohibitive. Involvement of more than one segment often presents with multiple regurgitant jets necessitating the need for multiple clips and this can be limited by both residual valve area and gradient. Chronic MR also leads to more prominent clefts between the leaflet scallops and this may make results with TEER less favorable. These indentations may form cleft like lesions that can lead to residual MR after grasping. Lastly myxomatous leaflets have abnormal cellular matrix that has not been considered desirable for grasping and this may perhaps lead to inferior results in MR therapy. Despite above noted challenges several groups have reported successful outcomes of TEER in patients with Barlow’s disease. Several maneuvers have been proposed to aid in leaflet grasping. The most relevant strategy involves anchoring an extremely flail segment by initially placing a clip adjacent to the flail segment/gap thus decreasing the flail gap. The initial clip acts as an anchor to allow stabilization of the second clip that is intended to grasp most of the flail segment [69, 70]. Other strategies that may lead to successful grasping in challenging pathologies include the use of positive end expiratory pressure support to decrease pre-load resulting in a decrease in flail gap size by decreasing the antero-posterior diameter of the mitral annulus [71].

Mitral valve clefts are indentations that are found in between scallops. Most true clefts occupy more than half the leaflet body and usually starts from leaflet tip to base. The use of 3D TEE has led to more accurate diagnosis of such pathology. The presence of clefts has been associated with residual MR after TEER [72]. Some proposed techniques to ensure less residual MR in the presence of a mitral cleft include deployment of MitraClip with a diagonal plane orthogonal to the coaptation plane. Other successful reports advocate consideration of the convergent clip technique [73]. This approach was described by Taramasso *et al*. [73] and suggests that an A-frame with the lateral clip orientation aligned more clockwise and the medial clip oriented in a more counterclockwise direction may serve patients with mitral valve clefts better results.

Another challenging group of patients include patients with medial or commissural pathology. In this patient profile more transseptal height is required to allow technical room for medial lesions. Anatomically there is higher risk of entanglement with chords and sub valvular apparatus. In the event of deployment of one clip a potential strategy to consider would be the deployment of an oblique clip that traverses different scallops may be considered, i.e., A1-P3 tissue bridge.

Lastly, a unique group of patients that have been of interest to TEER are patients with Hypertrophic Cardiomyopathy (HCM) and Systolic anterior mitral leaflet motion (SAM) causing Left ventricular outflow tract (LVOT) obstruction. Mitral plication therapy has been the standard approach to managing patients with HCM that are symptomatic and are deemed to be unfavorable candidates for septal alcohol ablation or surgical septal myectomy. The first series of patients were reported by Sorajja *et al*. [74] and this report showed both safety and efficacy in 5 patients with symptomatic LVOT obstruction. The approach to patients with HCM involves grasping A2-P2 leading to a midline tissue bridge with a reduction in displacement of the elongated anterior mitral leaflet into the LVOT. Follow up of such patients up to 19 months showed sustained reduction in LVOT gradient over time.

## 8. Failed prior surgical repairs

Surgical mitral valve repair may eventually fail, even when performed at experienced centers of excellence for valve disease [75, 76]. The rate of recurrence at 10 years of moderate to severe MR post-surgical repair is between 10% and 35%. Patients with anterior and bileaflet pathology have the highest risk of recurrence [77, 78, 79, 80].

The recurrence of MR post surgical repair has created a gap in the management of this unique patient population. Patients post surgical repair generally present at an older age given the durable nature of the surgical repair. Redo sternotomies are high risk and are associated with worse outcomes. This has suggested immense clinical need for alternative access and transseptal methods to treat post surgical MR. Current approaches include either a transcatheter mitral valve in ring or transcatheter edge-to-edge repair in ring. Some of the challenges with a valve in ring approach lies in the potential to cause left ventricular outflow tract obstruction or in the presence of a residual paravalvular leak post deployment.

**Fig. 7.**
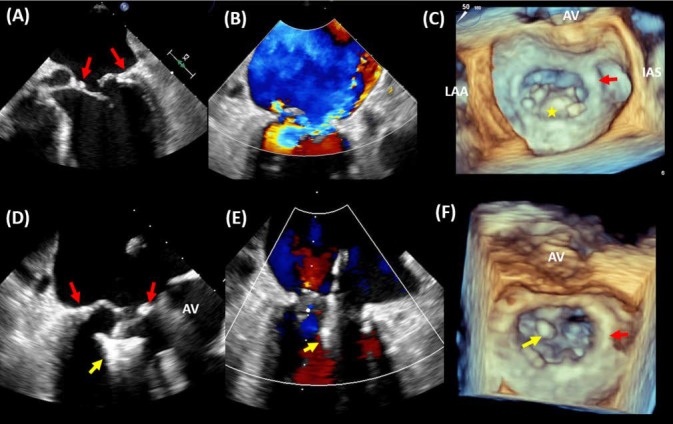
**MitraClip in annuloplasty ring**. A case of transcatheter edge-to-edge repair (TEER) in a patient with a surgical mitral valve ring (MVr). (A) Transesophageal echocardiography (TEE) showing a prolapsed posterior leaflet within the MVr (red arrow) resulting in severe eccentric MR (B). 3D TEE imaging demonstrating prolapsed posterior leaflet (*) within the MVr (C). The patient underwent TEER with MitraClip device (yellow arrow) implantation at A2-P2 (D) resulting in mild residual MR (E). 3D TEE imaging demonstrating the location of the MitraClip at A2-P2 in relation to the MVr (F). AV, aortic valve; LAA, left atrial appendage; IAS, interatrial septum.

This has led to the rational of TEER in ring. Emerging data has shown this approach to be a feasible option to treat post annuloplasty MR recurrence [81, 82, 83, 84]. There are however some technical considerations that pose challenges in this cohort of patients. First, the leaflet resection performed at the time of surgery may leave insufficient posterior leaflet length for a secure and stable grasp. A MitraClip NT device may be the device of choice in such situations, where grasping of 6 mm of leaflet length may be the only feasible option after surgery. Some reports have demonstrated an alternative technique of grasping the anterior leaflet with the posterior aspect of the annuloplasty ring when sufficient tissue length hinders leaflet grasping as a viable option [85]. Second, the mitral valve area after surgery may be small resulting in elevated diastolic gradients and significant mitral stenosis if a device is implanted. The last challenge to TEER in ring is embedded in the ability to obtain high resolution images appropriate for leaflet visualization and grasping. Standard mitral views may be difficult in the presence of acoustic shadowing that is likely to be present with a mitral annuloplasty ring. Some suggested approaches include off-axis imaging and the use of X-plane imaging frequently to overcome areas of shadowing. The use of 3D imaging for orientation and of clip descent is also key in ensuring successful grasp with minimal manipulation. If TEE proves to be challenging despite all the above, intracardiac echocardiography may be an alternative tool for guidance [86]. Some of the important parameters to obtain in a patient post mitral annuloplasty include mitral valve area, mead diastolic gradient, posterior leaflet length and detailed 2D and 3D imaging of both residual free edges at the site of propsed grasp. Fig. 7 demonstrates recurrent MR post mitral annuloplasty with successful Mitra Clip in ring implantation.

## 9. Outcomes post transcatheter edge-to-edge repair

Recent publications of real world follow up registries were revealing of promising of long-term follow up results related to TEER [87, 88, 89, 90]. Two of the largest registries with extended follow up include the MitraSwiss registry and the GIOTTO registry. Both European registries have over 1000 patients enrolled in each cohort. The MitraSwiss registry enrolled 1212 patients with both primary and secondary MR and reported acute procedural success rate of 91.5% [91]. Acute procedural success did not differ between a primary or secondary etiology of MR. Interestingly at 5 year follow up patients with degenerative MR had lower mortality and major adverse cardiac events. This observed outcome was not necessarily related to difference in MR pathology. This was likely due to the inherent baseline characteristics’ that patients in this population presented with. Patients with primary MR were older with few co-morbid conditions while patients with functional MR had a reduced ejection fraction, renal disease and anemia, all of which were independent predicters of mortality.

The GIOTTO registry is a multicenter prospective registry that reported outcomes of TEER from ten Italian centers. They included 1659 patients with functional and degenerative MR [92]. In their follow up, patients with functional MR were reported to have higher one- and two-year mortality. In their cohort of patients with degenerative mitral regurgitation, patients with 3+/4+ residual MR demonstrated worse outcomes. The presence of +1 residual MR in both cohorts was associated with improved survival.

Gavazzoni *et al*. [93] recently published a retrospective analysis of 69 patients with Barlow’s disease who had undergone TEER and were compared to 69 patients with flail or prolapse without features of Barlow’s disease. In this Swiss cohort overall procedural success rate was high in both groups. The number of clips used was higher in patients with Barlow’s disease and residual MR was also more significant compared to the non-Barlow patient group. The authors reported three-year outcomes of their patient cohort. The persistence of procedural results was more sustained in 80% in the non-Barlow disease group in comparison to 62% in the Barlow’s disease patient group. Subsequent mitral valve repair or replacement was seen in 10% of patients with Barlow’s disease compared to 5.7% of patients without the disease. Overall mortality was not stastically significant amongst the two groups, however there was a trend of increased heart failure related hospitalization in patients with Barlow’s disease. Real world TEER registry data suggests promising outcomes with durable results in a diverse patient population.

## 10. Devices under investigation

Another TEER system that is currently undergoing safety and effectiveness assessment is the PASCAL (Edwards Life Sciences, Irvine, CA, USA). The PASCAL system is currently being evaluated in the ongoing CLASPIID/IIF Pivotal Clinica trial . This safety and effectiveness trial aims to compare safety and outcomes of the PASCAL to MitraClip in patients with primary and secondary MR.

The PASCAL system has demonstrated safety effectiveness in a population patients degenerative, functional and mixed MR in the CLASP study. One- and two-year outcomes of the CLASP study showed a high rate of survival with a significant rate of reduction in heart failure related hospitalization. Significant MR reduction with positive LV remodeling was also appreciated as well as sustained improvement in patient functional status and exercise capacity [94, 95, 96]. This data led to Conformite Europeenne (CE) mark approval for the treatment of MR in Europe and the CLASP system has been in clinical use.

The PASCAL design has multiple similarities to the MitraClip delivery system with a guide sheath, steerable sheath and implant catheter. The device subtypes include the PASCAL and PASCAL Ace implant [97, 98]. The two subtypes differ in width with PASCAL being 10 mm wide and PASCAL Ace having a width of 6 mm. The configuration of the device is similar to the MitraClip consisting of paddles, clasp and a central spacer. The paddles function in a manner that allows them to promote leaflet approximation. The clasps similar to MitraClip have a primary function to ensure leaflet grasp. Another similarity to MitraClip include ability to grasp and maneuver independent of one another. Some of the differences between the to TEER devices are in the number of grippers. The CLASP has one row of grippers and MitraClip has between four to six. There are two unique elements to the PASCAL system. The presenc of a central spacre has been proposed to decrease tension on the leaflets and fill the occupy the regurgitant orifice. The second feature lies in the ability of the PASCAL to elongate inside the ventricle to promote safe retraction from the subvalvular apparatus and reduce risk of damaging the chords.The deployment strategy of the PASCAL system follows the same procedures as deployment of the MitraClip.

Two minimally invasive mitral annuloplasty techniques are under investigation. The NeoChord (Neochord Inc., St Louis Park, MN, USA) is currently in being studied in patients with primary degenerative disease that involve a flail or severely prolapsing segment. This device has demonstrated better efficacy in patients that have a more midline leaflet pathology involving a P2 segment [99, 100, 101, 102, 103, 104]. The RECHORD trial [105] is an ongoing prospective, multicenter, randomized FDA pivotal trial that aims to establish the safety and effectiveness of the device as an alternative to standard surgical mitral valve repair. The other device under trial is the HARPOON (Edwards Life Sciences).This also uses a mini-thoracotomy to reduce the degree of MR in patients with severe degenerative MR caused by posterior mitral leaflet prolapse by delivering and anchoring e-polytetrafluoroethylene (ePTFE) chords to the prolapsed mitral valve leaflet in a beating heart. The RESTORE IDE pivotal trial is being initiated in North America to evaluate the safety and effectiveness of the HARPOON MVRS in patients with severe degenerative MR presenting with mid-segment posterior mitral leaflet prolapse [106].

## 11. Conclusions

Transcatheter mitral valve repair in primary mitral regurgitation has altered the trajectory of patients deemed to be at a high or prohibitive risk for surgical intervention. Advancements in echocardiographic imaging, especially with 3D imaging, have facilitated appropriate patient selection and intraprocedural guidance in patients undergoing TEER. Alternative transcatheter mitral valve repair techniques for primary MR are emerging and are currently pending investigation in clinical trials.
